# Dipeptidyl peptidase-4 is associated with myogenesis in patients with adolescent idiopathic scoliosis possibly via mediation of insulin sensitivity

**DOI:** 10.1186/s13018-022-02978-w

**Published:** 2022-02-09

**Authors:** Zhicheng Dai, Bingchuan Xue, Leilei Xu, Zhenhua Feng, Zhichong Wu, Yong Qiu, Zezhang Zhu

**Affiliations:** 1 Department of Spine Surgery, Nanjing Drum Tower Hospital Clinical College of Nanjing Medical University, Zhongshan Road 321, Nanjing, 210008 China; 2 Department of Spine Surgery, Drum Tower Hospital of Nanjing University Medical School, Nanjing, China

**Keywords:** Adolescent idiopathic scoliosis, Dipeptidyl peptidase-4, Insulin sensitivity, Metabolism, Signaling

## Abstract

**Background:**

Abnormal metabolic features have been previously described in adolescent idiopathic scoliosis (AIS) patients. As an important regulator involved in energy metabolism, DPP-4 activity was reported to be remarkably decreased in osteoblasts of AIS patients. To date, there was still a lack of knowledge concerning the role of DPP-4 in the myogenesis of AIS.

**Methods:**

Circulation DPP-4 level was assessed in the serum of 80 AIS girls and 50 healthy controls by ELISA. Myoblasts were purified from muscle specimens of AIS patients and LDH controls, and then treated with metabolic effectors including glucose and insulin. CCK-8 assay was used to assess the cell viability and myotube fusion index was calculated to evaluate myogenesis ability. Gene expressions of downstream signals of DPP-4 were evaluated by RT-qPCR and Western blot respectively.

**Results:**

AIS girls had remarkably down-expressed DPP-4 in both serum level (0.76 fold) and tissue (0.68 fold) level. Treatment with metabolic effectors led to significantly increased DPP-4 expression in the control cells, while there was no increase of DPP-4 in AIS cells. CCK-8 assay showed that the proliferation rate of control cells was significantly increased after being treated. Remarkably higher fusion index was also observed in the treated control cells. By contrast, the fusion index and cell proliferation rate were comparable between the treated and the untreated AIS cells.

**Conclusions:**

Our study suggested a potential role of DPP-4 in abnormal metabolic condition of AIS patients. Compared with control cells, AIS myoblasts presented obviously impaired sensitivity to the treatment of glucose and insulin. Aberrant DPP-4 expression could lead to impaired insulin sensitivity in myoblasts and further influence the cell viability during myogenesis. The molecular mechanism connecting DPP-4 and insulin-related signaling in AIS is worthy of further investigation.

**Supplementary Information:**

The online version contains supplementary material available at 10.1186/s13018-022-02978-w.

## Introduction

Adolescent idiopathic scoliosis (AIS) is a 3-dimensional deformity which occurs during the pubertal growth [1]. To date, there is no consensus on the etiology of AIS. Many factors were proposed to be involved in the development of AIS, including genetic variation, metabolism dysfunction, and abnormal neuromuscular function [2–4]. It has been well documented that AIS patients tended to have different anthropometric parameters compared with age-matched controls, such as taller stature with lower body mass index (BMI) and lower bone mineral density (BMD), while the cause of these differences remains obscure [5, 6]. Since there was no significant difference of nutrition intake between AIS girls and controls, it was therefore speculated that the abnormal energy homeostasis including appetite regulation, energy expenditure and insulin sensitivity might be impaired in AIS girls [7]. Several hormone and metabolic molecule, such as leptin [8], melatonin [9, 10], and lipid metabolite [11] have been revealed to be associated with AIS.

Dipeptidyl peptidase-4 (DPP-4) was a type of transmembrane protein that can modulate insulin-related metabolism [12]. DPP-4 inhibitor, launched and wildly used in clinical practice, was proven to improve BMD and decrease the risk of fracture in type 2 diabetes, establishing a relationship between DPP-4 and bone quality [13]. Normand et al. [14] observed lower serum expression of DPP-4 in AIS patients. Moreover, remarkable differences in DPP-4 activity and regulation between osteoblasts of AIS patients and healthy controls were found in their study.

Except for decreased bone density, AIS patients also presented lower body mass and abnormal growth pattern of paraspinal muscles. Previous studies have shown that DPP-4 could modulate muscular energy homeostasis, insulin secretion and sensitivity directly, which may further affect muscle proliferation and differentiation in adolescents [15–17]. However, to our knowledge, there was a lack of study investigating the role of DPP-4 in the modulation of myogenesis in AIS patients. In the current study, we aimed to investigate the influence of DPP-4 on the biological viability of myoblasts and to further characterize its role in the metabolic condition of AIS.

## Methods

### Subjects

The inclusion criteria were shown as follows: 1. female; 2. diagnosed as AIS through clinical and radiological examinations; 3. with single thoracic curve. Age-matched female lumbar disc herniation (LDH) patients and healthy girls were also enrolled in this study. All the AIS and LDH patients came to our clinic center for surgery treatment, and the healthy controls were recruited through a routine physical examination program for adolescent students during May 2013 and November 2018. Scoliosis patients secondary to trauma, tumor, neuromuscular deficit and infection were excluded. The baseline characteristics were recorded, including age, body mass index (BMI), bone mineral density (BMD) and curve magnitude at the first visit.

### Sample collection

Blood samples were collected from 50 healthy girls and 80 AIS patients. All the blood samples were treated with sodium citrate, centrifuged to isolate serum, and stored at − 80 °C until thawed and analyzed. Paravertebral muscles of the proximal vertebrae were collected from 45 AIS and 30 LDH patients during surgery with the informed consent obtained from their parents. Deep paraspinal muscle biopsies of 1.5 × 1.5 × 1.5cm^3^ were stored at − 80 °C directly.

### Enzyme-linked immunosorbent assay (ELISA)

ELISA analysis of serum DPP-4 was conducted according to the protocol previously described by guidebook of CD26 Human ELISA kit (Abcam, Cambridge, UK). Assays were performed according to manufacturer’s protocol and samples were analyzed without dilution.

### Myoblast isolation and purification

Paraspinal muscle of 3 AIS patients and 3 LDH controls were randomly selected for digestion to single cell suspension, treated with mixed enzyme containing Collagenase D (0.15 U/mg, Sigma, Darmstadt, Germany) 10 mg/mL, Dispase II (0.5 U/mg, Sigma, Darmstadt, Germany) 4.8 mg/mL, and 250 mM CaCl_2_ 50 mM/mL. After centrifugation, cells were resuspended in growth media containingF12 (Gibco) complemented with 20% FBS (Gibco), 1% P/S (Gibco), 1% α-glutamine (Sigma), and 2.5 ng/mL FGF (Sigma). The culture medium was changed every 2 days. After 7–10 days, when cells reached 70–80% confluence, the purification of myoblast was conducted with immunomagnetic beads technology following the manufacture instructions of CD56 MicroBeads (Miltenyi, Gladbach, Germany). After 3–5 days of cell proliferation, the purity of myoblasts was then identified by immunofluorescence (Additional file 1, 2: Figs. S1, S2).

### Immunofluorescence microscopy

The myoblasts were blocked by being incubated in PBS supplemented with 2% goat serum (block solution) for 30 min. After removal of the block solution, the samples were treated with primary antibody rabbit anti-desmin (1:200; Abcam), mouse Anti-MHC antibody (1:50; Developmental Studies Hybridoma Bank) and incubated overnight at 4 °C in a humidified chamber. The sections were washed with PBS and then incubated for 1 h in a dark chamber with Alexa Fluor 594-conjugated Affinipure Goat Anti-Mouse IgG (H + L) (1:500; Jackson ImmunoResearch) and Alexa Fluor 488-conjugated Affinipure Goat Anti-Rabbit IgG (H + L) (1:500, Jackson ImmunoResearch). DAPI (Thermo fisher scientific, Massachusetts, USA) was used to seal slides for 4 min in the dark. Images of stained cells were taken using a fluorescence microscope (Axio Observer, Zeiss, Oberkochen, Germany). 3 slides were observed in each case, and positive-staining cell was counted via ImageJ software.

### Cell culture and metabolic effector treatment

Myoblasts were cultured in growth medium consisting of F-12 (Gibco)with 20% FBS and 1% penicillin–streptomycin at 37 °C. To evaluate glucose metabolism in myoblasts, 0.5 nM insulin (Beyotime, Shanghai, China) and 10 mM glucose (Sigma-Aldrich, Oakville, ON, Canada) were added to the wells after a 24 h pre-incubation with serum-free F-12 media. Cell viability was assessed using the CCK-8 assay, for each sample, the cells incubated only with growth medium were used as the negative control.

To induce myogenic differentiation, the culture medium was switched to F12 (Gibco) supplemented with 2% horse serum (Gibco), 1% P/S (Gibco), 1% α-glutamine (Sigma). Myoblasts from AIS and control group were induced to differentiation added with metabolic effectors for 5 days, respectively. Cells incubated without glucose and insulin were used as the negative control. Fusion index was used to assess the formation of myotube as previously reported [18].

### RNA extraction and real time-polymerase chain reaction (RT-qPCR) analysis

Total RNA was extracted from tissue and myoblasts with TRIzol reagent (Invitrogen). Gene expression levels were quantitated using SYBR Master Mix (TAKARA, Tokyo, Japan) on a Light Cycler 480 (Roche Applied Science, Mannheim, Germany) with glyceraldehyde 3-phosphate dehydrogenase (GAPDH) as the endogenous control. The amplification protocol included an initial denaturation step at 95 °C for 10 min, followed by 44 cycles of denaturation at 95 °C for 10 s, annealing at 60 °C for 20 s and elongation at 72 °C for 10 s. At the end of the cycling protocol, melting curve analyses were performed. Relative mRNA expression was analyzed based on the 2^−∆∆Ct^ method. The primers were as follows: 5′-GCTCGGCGCTCACTAATGTT-3′ (forward) and 5′-AGAACCTTCCACGGTGTCTTC-3′ (reverse)for DPP-4; 5′-CAGCTTGACTCAAAATTCCTGGA -3′ (forward) and 5′-TGAAGATTACGCTTGCTTTTCCT -3′ (reverse) for STAT1; 5′-GGAGCGAGATCCCTCCAAAAT-3′ (forward), and 5′-GGCTGTTGTCATACTTCTCATGG-3′ (reverse) for GAPDH.

### Protein isolation and Western blot (WB) analysis

Tissues and myoblasts were lysed on ice in RIPA buffer (1 × phosphate-buffered saline, 1% NP40, 0.1% SDS, 5 mM ethylenediaminetetraacetic acid, 0.5% sodium deoxycholate and 1 mM sodium orthovanadate) treated with protease inhibitor (Complete ethylenediaminetetraacetic acid-free; Roche). Equal amounts of lysis supernatant were fractionated by 8–10% SDS–polyacrylamide gel electrophoresis for immunoblot analysis. Separated proteins were then transferred to a polyvinylidene difluoride membrane (Immobile P; Millipore), which was then exposed to 5% dried skim milk in a solution containing 50 mM Tris–HCl (pH 7.5), 150 mM NaCl, and 0.1% Tween 20 (TBST). Membranes were incubated with primary antibodies overnight at 4 °C and then with corresponding secondary antibody conjugated to horseradish peroxidase for 1 h at room temperature. Primary antibodies were listed as follows: DPP-4 (1:2000; ab28340, Abcam), Ras (1:5000; ab52939, Abcam), p-ERK1/2 (1:5000; ab201015, Abcam), p-mTOR (1:1000; #5536, Cell signaling technology), p-AKT (1:2000, #4060, Cell signaling technology) and GAPDH (1:2000; #5174, Cell signaling technology). The signals were detected by enhanced chemiluminescence.

### Statistical analysis

After being tested for normality, all the data were observed to conform to Gaussian distribution. Continuous variables were shown as mean ± standard deviation (SD) and were analyzed via SPSS version 20.0 (SPSS, Chicago, IL, USA). Intergroup comparison between AIS group and the control group was compared with the Student's *t* test. The linear relationship between different variables, including gene expression, BMD, and BMI, was analyzed by the Pearson correlation analysis. Statistically significant difference was set at *p* < 0.05.

## Results

### Baseline characteristics of the subjects

For ELISA analysis, the average age for AIS patients and healthy girls was 13.9 ± 2.5 years and 14.3 ± 3.3 years (*p* = 0.44), respectively. The average BMI was 17.9 ± 2.3 kg/m^2^ for AIS patients and 18.5 ± 3.0 kg/m^2^ for healthy girls (*p* = 0.20).

For tissue expression analysis, as shown in Table 1, the mean age was 15.4 ± 2.7 years for AIS patients and 15.8 ± 4.2 years for controls (*p* = 0.32). The mean BMI was 18.6 ± 1.6 kg/cm^2^ for AIS patients and 19.8 ± 4.5 kg/cm^2^ for controls(*p* = 0.10). The mean curve magnitude was 49.3 ± 5.4 degrees. The mean Bone mineral density (BMD) of the AIS patients was 0.84 ± 0.10 g/cm^2^.

**Table 1:** Baseline clinical characteristics of the subjects for gene expression analysis

Variables	AIS patients (*n* = 45)	LDH controls (*n* = 30)
Age (yrs)	15.4 ± 2.7	15.8 ± 4.2
Body mass index (kg/m^2^)	18.6 ± 1.5	19.8 ± 4.5
Bone mineral density (g/cm^2^)	0.84 ± 0.10	NA
Cobb angle (°)	49.3 ± 5.4	NA

### Expression of DPP-4 and STAT1 in serum and paraspinal muscles

**Fig. 1:**
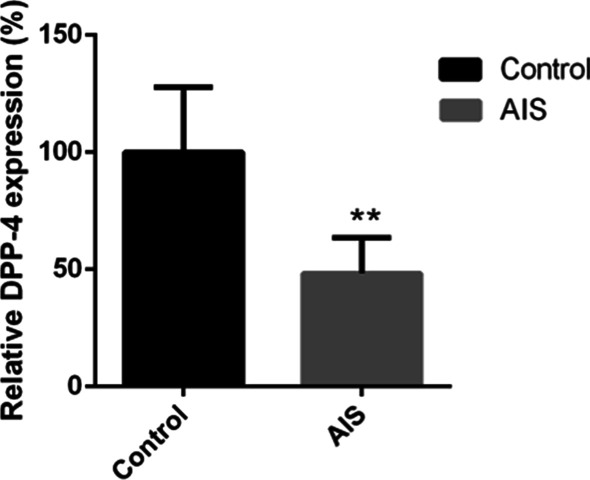
Serum DPP-4 level in AIS patients and healthy controls. The serum DPP-4 activity was remarkably lower in AIS patients than in the healthy controls. *p < 0.05 using two-tailed Student’s *t*-test. Data are presented as mean ± standard deviation

**Fig. 2:**
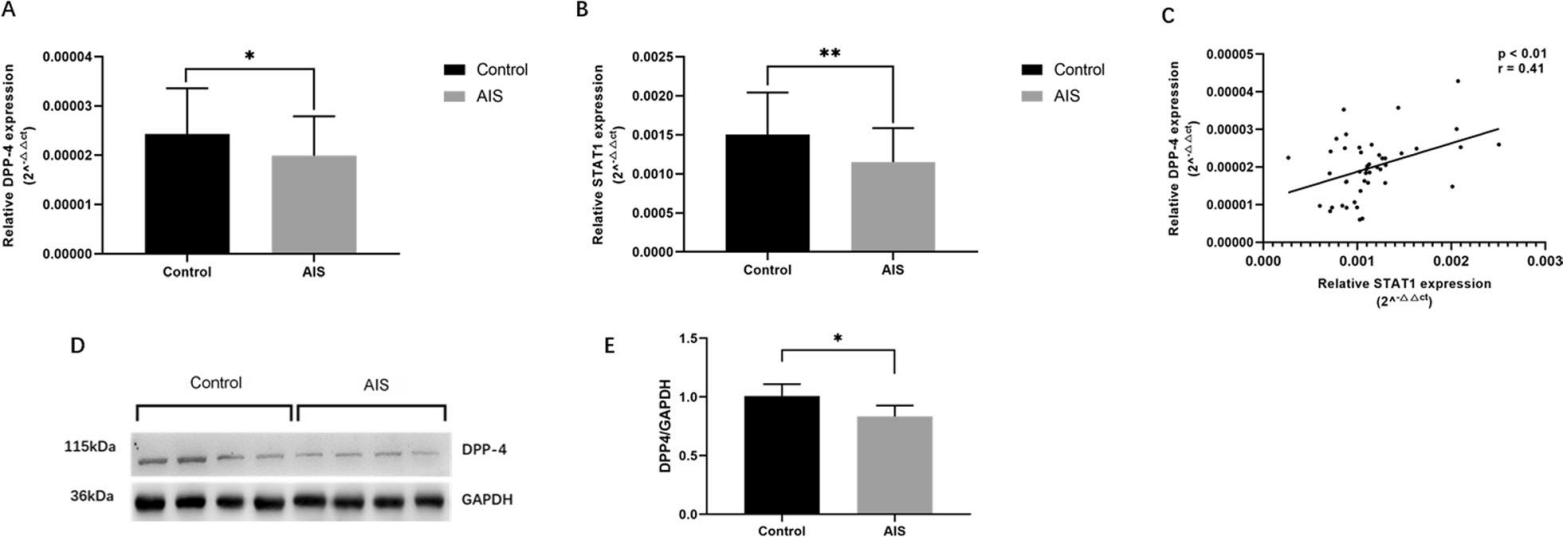
DPP-4 gene and protein expression in muscle tissue isolated from LDH patients and AIS patients. **A**, **B** Relative expression of DPP4 and STAT1 was significantly decreased in patients (*n* = 45) as compared with that of the controls (*n* = 30). **C** mRNA expression level of DPP4 gene were strongly correlated with STAT1 mRNA expression level (*r* = 0.41, *p* < 0.05). Pearson’s correlation analysis was used to determine the correlation. **D**, **E** Western blot showed DPP-4 protein expression in muscle was significantly higher in controls (*n* = 4) as compared with AIS patients (*n* = 4). *p < 0.05, two-tailed Student’s *t* test. Data are presented as mean ± standard deviation

The serum DPP-4 activity was remarkably lower in AIS girls than in the healthy controls (*p* < 0.05) (Fig. 1). Tissue expression analysis showed that the mRNA expression of DPP-4 and STAT1 was significantly decreased in patients as compared with that of the LDH controls (0.000020 ± 0.000008 vs. 0.000024 ± 0.000009, *p* = 0.032 for DPP-4; 0.001151 ± 0.000436 vs. 0.001504 ± 0.000538, *p* = 0.003 for STAT1) (Fig. 2A, B). Significant correlation between the expression of DPP-4 and STAT1 in paraspinal muscles was observed (*r* = 0.41, *p* = 0.005) (Fig. 2C). DPP-4 protein expression in muscle was significantly higher in controls as compared with AIS patients (Fig. 2D, E).

### The influence of metabolic factors on DPP-4 expression

**Fig. 3:**
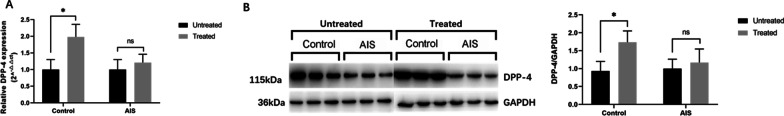
Impact of treatment with glucose and insulin on DPP-4 expression. Cells were treated with glucose (10 mM) and insulin (0.5 nM) for 24 h. **A** The mRNA expression of DPP-4 was significantly increased in the treated control cell as compared with the untreated control cells (*n* = 3/group). **B** The DPP-4 protein level was significantly increased in the treated control cells as compared with the untreated control cells (*n* = 3/group). No significant difference of DPP4 mRNA or protein expression level was observed between the treated and untreated cells in AIS. *p < 0.05, two-tailed Student’s *t* test, two-tailed Student’s *t* test. Data are presented as mean ± standard deviation

With the treatment for 24 h. The mRNA expression of DPP-4 was increased by 2.38 folds in the treated control cells as compared with the untreated control cells (*p* < 0.01) (Fig. 3A). The DPP-4 protein level increased 1.8 times more in the treated control cells. By contrast, for the AIS group, there is no significance between the treated and the untreated cells (*p* > 0.05) (Fig. 3B).

### The effect of metabolic factors on myoblast differentiation and viability

**Fig. 4:**
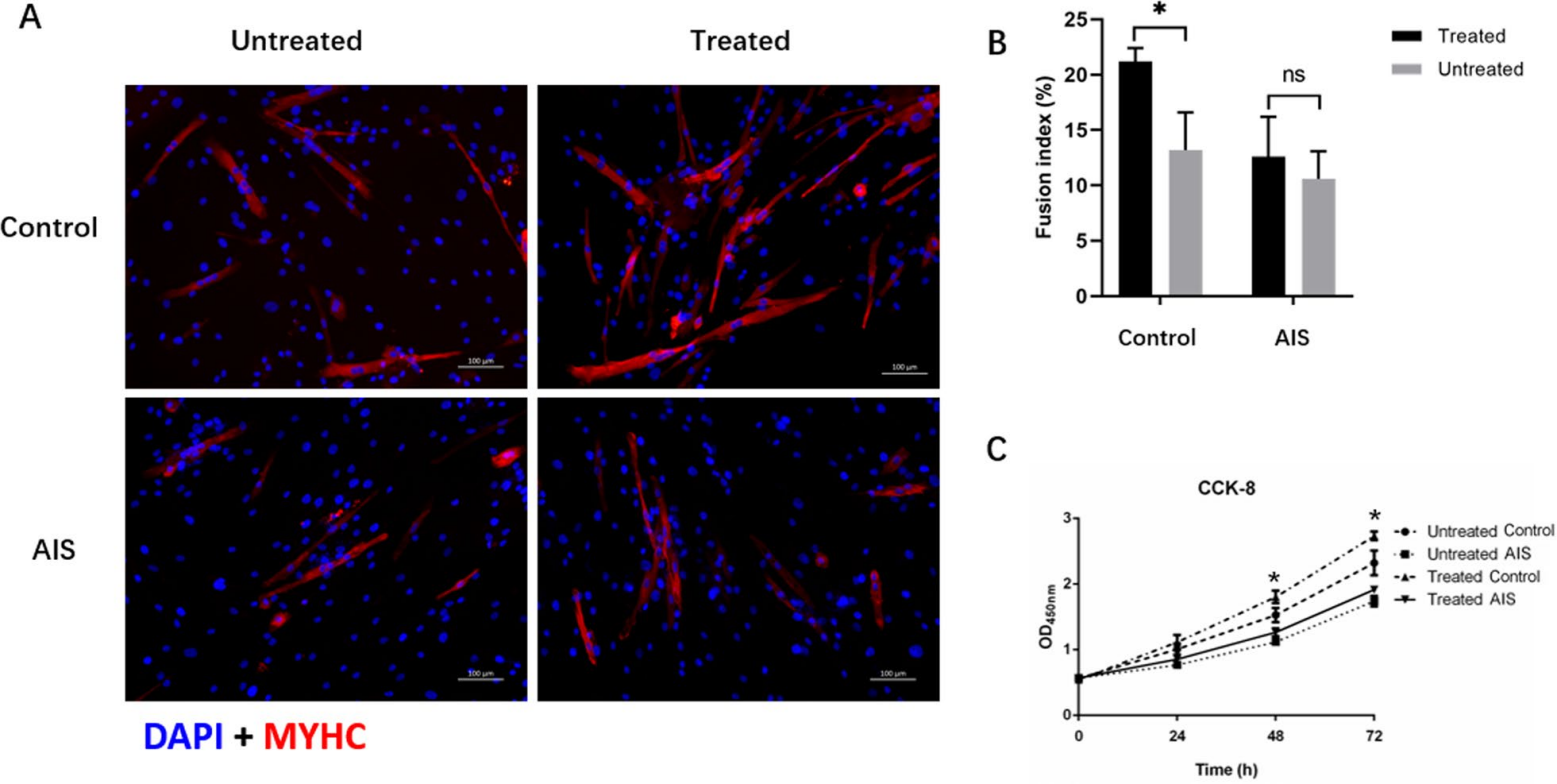
The effect of treatment with glucose and insulin on myoblast proliferation and differentiation ability in AIS and control group. **A** Primary myoblast of AIS and control group were induced to differentiation for 5 days and stained for Myosin heavy chain (MyHC) and a nuclear stain (DAPI). Remarkably higher fusion index was observed in the treated control cells as compared with the untreated cells in the control group (*n* = 3/group). **B** Remarkably higher fusion index was observed in the treated control cells as compared with the untreated cells in the control group (*n* = 3/group). **C** The impact of effectors on myoblasts proliferation was assessed using the CCK-8 assay. Treatment of effectors remarkably increased cell viability of myoblast in controls (*n* = 3/group). **p < 0.01, paired Student’s *t* test. Data are presented as mean ± standard deviation

Remarkably higher fusion index was observed in the treated control cells as compared with the untreated cells in the control group (Fig. 4A, B). By contrast, the fusion index was comparable between the treated cells and the untreated cell in the AIS group (Fig. 4A, B). CCK-8 assay showed that the proliferation rate of control cells was significantly increased after being treated. As for AIS cells, no significant increase of the proliferation rate was observed after being treated (Fig. 4C).

### Regulation of Ras/ERK and Akt/mTOR pathway by metabolic effectors

**Fig. 5:**
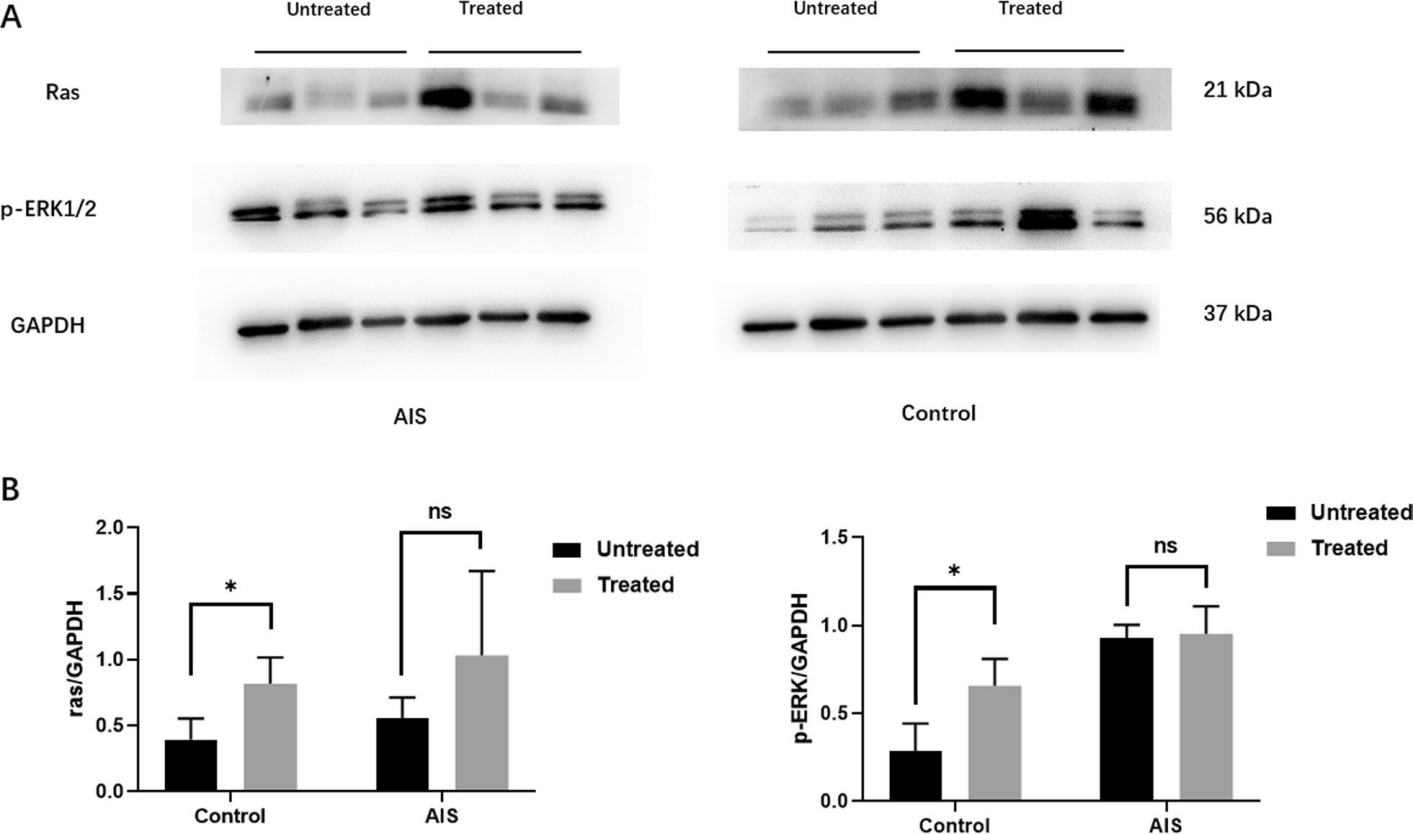
Impact of treatment with glucose and insulin on Ras/ERK pathway. The Ras protein and p-ERK1/2 expression was significantly elevated in the treated control cells. No significant difference regarding Ras/p-ERK expression was observed between the treated and the untreated AIS cells (*n* = 3/group). *p < 0.05, two-tailed paired Student’s *t* test. Data are presented as mean ± standard deviation

To determine the influence of metabolic effectors on glucose metabolism signaling pathway, protein expression of Akt/mTOR and Ras/ERK was evaluated by WB. As illustrated in Fig. 5A, B, for Ras/ERK pathway, the Ras protein and ERK phosphorylation expression were significantly elevated in the treated control cells, (*p* < 0.05). By contrast, there was no significant difference regarding Ras/ERK expression between the treated and the untreated AIS cells. As shown in Additional file 2: Fig. 2, for Akt/mTOR pathway, the Akt phosphorylation and mTOR phosphorylation were comparable between the treated cells and the untreated cells in both AIS group and the control group (*p* > 0.05).

### Relationship between DPP-4 and clinical features of AIS

**Fig. 6:**
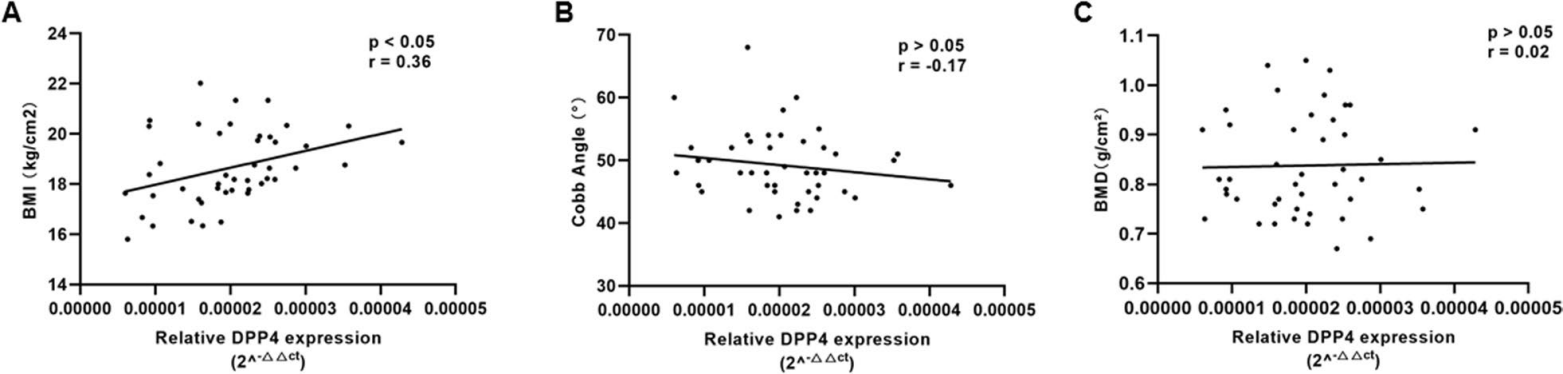
Relationship between DPP-4 and clinical features of AIS. DPP4 gene expression in muscle tissue was measured by RT-qPCR and analyzed with the 2^−ΔΔCT^ method (*n* = 45). **A** There was a significant correlation between the expression level of DPP-4 and BMI in AIS patients. **B** There was no significant correlation between DPP4 expression and curve severity. **C** No significant correlation was observed between DPP-4 expression and BMD

As shown in Fig. 6, there was a significant correlation between the expression level of DPP-4 and BMI in AIS patients (*r* = 0.36, *p* = 0.01). However, such linear relationship was not observed between DPP-4 expression and curve severity (*r* = − 0.17, *p* = 0.27) or between DPP-4 expression and BMD (*r* = 0.02, *p* = 0.89).

## Discussion

Energy metabolism played an important role in muscle development and function, and insulin acts as a significant effector in this procedure [19]. Several studies have reported that the circulating DPP-4 level was related to insulin resistance [20–22]. Daniela et al. [23] reported that the pathological release of DPP-4 in Type 2 diabetes might impair insulin pathway, especially insulin sensitivity. As documented in earlier literatures, abnormal metabolism has been implicated in the etiology of AIS. To characterize the role of DPP-4 in the metabolism of AIS patients, Normand et al. [14] firstly isolated osteoblast from AIS patients and controls and reported abnormally down-regulated expression of DPP-4 in AIS patients. In the current study, we successfully validated that AIS subjects had remarkably lower DPP-4 expression in both serum and paraspinal muscles when compared with the controls. It was known that STAT1 could regulate the expression of DPP-4 by binding to its promoter region as a transcriptional factor. In line with this research, we observed that the DPP-4 and STAT1 were simultaneously down-expressed in paraspinal muscles of AIS. Moreover, the expression of STAT1 was significantly correlated with that of DPP-4. It was therefore plausible that STAT1 may be involved in the abnormal expression of DPP-4 in AIS patients. It was worthwhile to investigate the molecular mechanism underlying the regulation of STAT1 on DPP-4 in future study.

As reported in earlier literatures, DPP-4 strongly correlates with obesity in normal cohort. Kirino et al. [24] reported that DPP-4 activity displayed strong positive correlations with BMI in healthy young people. Interestingly, DPP-4 expression in AIS subjects was found significantly correlated with BMI. Patients with lower BMI were found to have decreased circulating expression of DPP-4. As for BMD and curve severity, a lack of correlation with DPP-4 expression was noted. Based on these findings, it was likely that insufficient production of DPP-4could damage physiological energy metabolism in body mass, thereby involved in the development of AIS.

To further determine the role of DPP-4 in the etiology of AIS, we investigated the relationship between DPP-4 expression and insulin sensitivity in the patients. Insulin sensitivity was reported to play an important role in myogenesis. For the first time, we isolated myoblast from AIS patients and evaluated the influence of metabolic effectors on cell viability. A lack of response to the treatment was observed in AIS myoblasts as evidenced by the unchanged DPP-4 expression and cell viability. By contrast, remarkably higher expression of DPP-4 and increased cell viability in normal myoblasts was triggered by insulin and high glucose. Moreover, insulin and high glucose could promote myoblast differentiation in normal myoblast. On the contrary, differentiation potential was not changed after the treatment to AIS myoblast. These outcomes might reflect impaired insulin sensitivity in AIS patients resulted from aberrant regulation of DPP-4, which may in turn affected muscle growth and differentiation.

Ras/ERK pathway and Akt/mTOR pathway were fundamental for insulin-related signal transduction in cell proliferation and differentiation [25, 26]. In the current study, we observed significant divergence regarding expression of downstream signaling between AIS and normal cells after being treated by metabolic effectors. Remarkably increased expression of Ras/ERK was observed in normal myoblasts. By contrast, for AIS myoblasts there was no difference in the expression of Ras/ERK pathway. Moreover, the Akt/mTOR pathway were comparable between the treated cells and the untreated cells in both AIS group and the control group. Our findings partially uncovered the mechanism of impairing insulin-conducted signaling in AIS. Namely, deficiency of DPP-4 may be associated with altered insulin sensitivity, thus leading to down-expressed Ras/ERK signaling in AIS tissues. The mechanism connecting DPP-4 and Ras/ERK pathway is worthy of further exploration.

Based on above results, lower DPP-4 was associated with abnormal metabolism of musculoskeletal system in AIS, thus disturbing the homeostasis of spine and making adolescent more susceptible to scoliosis. We speculated that DPP-4 might not only affect metabolic pathway, but also contribute to the development of AIS.

Two limitations of the present study need to be addressed. The sample size of the control group enrolled in the tissue expression analysis was relatively small. Since it was difficult to obtain the muscles samples from healthy girls, we recruited age-matched female LDH patients and collected the paraspinal muscles during surgery, which may lead to potential bias. Tissues from patients with spine trauma can be collected in future study to further validate our findings. Second, we preliminarily investigated the role of DPP-4 in metabolism of AIS patients through expression analysis. More in-vivo experiments are warranted to further uncover the molecular mechanism underling the regulation of DPP-4 on insulin sensitivity of AIS patients.

## Conclusions

Our study suggested a potential role of DPP-4 in abnormal metabolic condition of AIS patients. DPP-4 expression level was down-regulated in patients with AIS. Aberrant DPP-4 expression could affect insulin sensitivity in myoblasts and further influence the cell viability during myogenesis. The molecular mechanism connecting DPP-4 and insulin-related signaling such as Ras/ERK pathway is worthy of further investigation.

## Supplementary Information

**Additional file 1: Fig. S1**. The purity of myoblasts identified by immunofluorescence. The percentage of Desmin positive cells was used to evaluate the purity of primary myoblasts isolated from AIS and LDH patients (*n* = 3/group). The purity was more than 90%.

**Additional file 2: Fig. S2**. Impact of treatment with glucose and insulin on AKT/mTOR pathway. The p-AKT and p-mTOR protein expression was comparable between the untreated and treated group (*n* = 3/group). ns, not significant. two-tailed paired Student’s *t* test. Data are presented as mean ± standard deviation.

## Data Availability

All data generated or analysed during this study are included in this published article [and its supplementary information files].

## Abbreviations

AIS: Adolescent idiopathic scoliosis
DPP-4: Dipeptidyl peptidase-4
LDH: Lumber disc herniation

## References

[1] Raso VJ, Lou E, Hill DL, Mahood JK, Moreau MJ, Durdle NG (1998). Trunk distortion in adolescent idiopathic scoliosis. J Pediatr Orthop.

[2] Cheng JC, Castelein RM, Chu WC, Danielsson AJ, Dobbs MB, Grivas TB, Gurnett CA, Luk KD, Moreau A, Newton PO (2015). Adolescent idiopathic scoliosis. Nat Rev Dis Primers.

[3] Haasbeek JF (1997). Adolescent idiopathic scoliosis. Postgrad Med.

[4] Maffulli N (1990). Histochemical and physiological studies in idiopathic scoliosis. Ital J Orthop Traumatol.

[5] Siu King Cheung C, Tak Keung Lee W, Kit Tse Y, Ping Tang S, Man Lee K, Guo X, Qin L, Chun Yiu Cheng J (2003). Abnormal peri-pubertal anthropometric measurements and growth pattern in adolescent idiopathic scoliosis: a study of 598 patients. Spine (Phila Pa 1976).

[6] Barrios C, Cortes S, Perez-Encinas C, Escriva MD, Benet I, Burgos J, Hevia E, Piza G, Domenech P (2011). Anthropometry and body composition profile of girls with nonsurgically treated adolescent idiopathic scoliosis. Spine (Phila Pa 1976).

[7] Lee WT, Cheung CS, Tse YK, Guo X, Qin L, Ho SC, Lau J, Cheng JC (2005). Generalized low bone mass of girls with adolescent idiopathic scoliosis is related to inadequate calcium intake and weight bearing physical activity in peripubertal period. Osteoporos Int.

[8] Tam EM, Liu Z, Lam TP, Ting T, Cheung G, Ng BK, Lee SK, Qiu Y, Cheng JC (2016). Lower muscle mass and body fat in adolescent idiopathic scoliosis are associated with abnormal leptin bioavailability. Spine (Phila Pa 1976).

[9] Morocz M, Czibula A, Grozer ZB, Szecsenyi A, Almos PZ, Rasko I, Illes T (2011). Association study of BMP4, IL6, Leptin, MMP3, and MTNR1B gene promoter polymorphisms and adolescent idiopathic scoliosis. Spine (Phila Pa 1976).

[10] Gargano G, Oliva F, Migliorini F, Maffulli N (2021). Melatonin and adolescent idiopathic scoliosis: the present evidence. Surgeon.

[11] Sun ZJ, Jia HM, Qiu GX, Zhou C, Guo S, Zhang JG, Shen JX, Zhao Y, Zou ZM (2016). Identification of candidate diagnostic biomarkers for adolescent idiopathic scoliosis using UPLC/QTOF-MS analysis: a first report of lipid metabolism profiles. Sci Rep.

[12] Matteucci E, Giampietro O (2009). Dipeptidyl peptidase-4 (CD26): knowing the function before inhibiting the enzyme. Curr Med Chem.

[13] Monami M, Dicembrini I, Antenore A, Mannucci E (2011). Dipeptidyl peptidase-4 inhibitors and bone fractures: a meta-analysis of randomized clinical trials. Diabetes Care.

[14] Normand E, Franco A, Moreau A, Marcil V (2017). Dipeptidyl peptidase-4 and adolescent idiopathic scoliosis: expression in osteoblasts. Sci Rep.

[15] Arnes L, Gonzalez N, Tornero-Esteban P, Sancho V, Acitores A, Valverde I, Delgado E, Villanueva-Penacarrillo ML (2008). Characteristics of GLP-1 and exendins action upon glucose transport and metabolism in type 2 diabetic rat skeletal muscle. Int J Mol Med.

[16] Bouchi R, Fukuda T, Takeuchi T, Nakano Y, Murakami M, Minami I, Izumiyama H, Hashimoto K, Yoshimoto T, Ogawa Y (2018). Dipeptidyl peptidase 4 inhibitors attenuates the decline of skeletal muscle mass in patients with type 2 diabetes. Diabetes Metab Res Rev.

[17] Baumeier C, Schluter L, Saussenthaler S, Laeger T, Rodiger M, Alaze SA, Fritsche L, Haring HU, Stefan N, Fritsche A (2017). Elevated hepatic DPP4 activity promotes insulin resistance and non-alcoholic fatty liver disease. Mol Metab.

[18] Millay DP, O'Rourke JR, Sutherland LB, Bezprozvannaya S, Shelton JM, Bassel-Duby R, Olson EN (2013). Myomaker is a membrane activator of myoblast fusion and muscle formation. Nature.

[19] Scheuermann-Freestone M, Madsen PL, Manners D, Blamire AM, Buckingham RE, Styles P, Radda GK, Neubauer S, Clarke K (2003). Abnormal cardiac and skeletal muscle energy metabolism in patients with type 2 diabetes. Circulation.

[20] Zhuge F, Ni Y, Nagashimada M, Nagata N, Xu L, Mukaida N, Kaneko S, Ota T (2016). DPP-4 inhibition by linagliptin attenuates obesity-related inflammation and insulin resistance by regulating M1/M2 macrophage polarization. Diabetes.

[21] Kusunoki M, Sato D, Nakamura T, Oshida Y, Tsutsui H, Natsume Y, Tsutsumi K, Miyata T (2015). DPP-4 inhibitor teneligliptin improves insulin resistance and serum lipid profile in Japanese patients with type 2 diabetes. Drug Res (Stuttg).

[22] Sa-Nguanmoo P, Tanajak P, Kerdphoo S, Jaiwongkam T, Pratchayasakul W, Chattipakorn N, Chattipakorn SC (2017). SGLT2-inhibitor and DPP-4 inhibitor improve brain function via attenuating mitochondrial dysfunction, insulin resistance, inflammation, and apoptosis in HFD-induced obese rats. Toxicol Appl Pharmacol.

[23] Lamers D, Famulla S, Wronkowitz N, Hartwig S, Lehr S, Ouwens DM, Eckardt K, Kaufman JM, Ryden M, Muller S (2011). Dipeptidyl peptidase 4 is a novel adipokine potentially linking obesity to the metabolic syndrome. Diabetes.

[24] Kirino Y, Sei M, Kawazoe K, Minakuchi K, Sato Y (2012). Plasma dipeptidyl peptidase 4 activity correlates with body mass index and the plasma adiponectin concentration in healthy young people. Endocr J.

[25] Coffer PJ, van Puijenbroek A, Burgering BM, Klop-de Jonge M, Koenderman L, Bos JL, Kruijer W (1997). Insulin activates Stat3 independently of p21ras-ERK and PI-3K signal transduction. Oncogene.

[26] Zoncu R, Efeyan A, Sabatini DM (2011). mTOR: from growth signal integration to cancer, diabetes and ageing. Nat Rev Mol Cell Biol.

