# Supplementary figures and images for: Dipeptidyl peptidase-4 is associated with myogenesis in patients with adolescent idiopathic scoliosis possibly via mediation of insulin sensitivity
Source: J Orthop Surg Res. 2022 Feb 9;17:82. doi: 10.1186/s13018-022-02978-w (PMC8827187; doi:10.1186/s13018-022-02978-w)

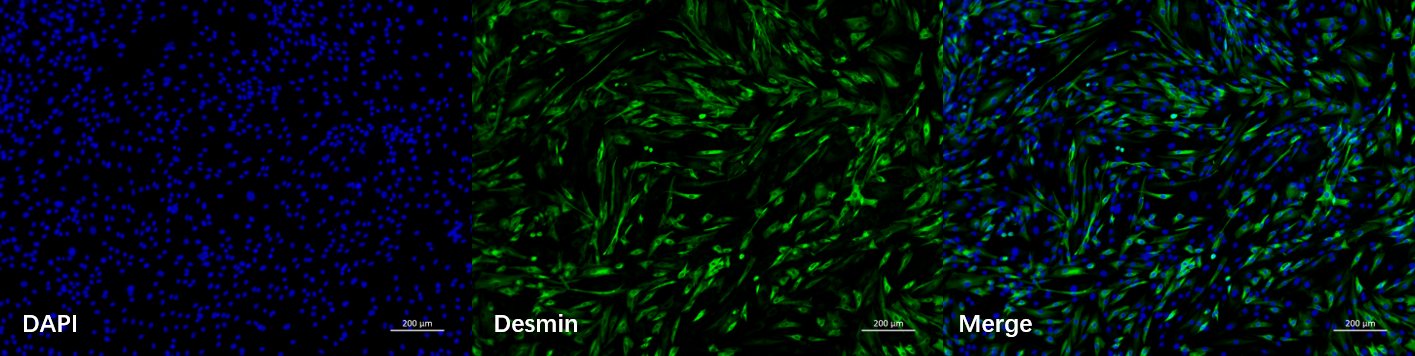

Supplement: Supplementary file 1 — Additional file 1: Fig. S1. The purity of myoblasts identified by immunofluorescence. The percentage of Desmin positive cells was used to evaluate the purity of primary myoblasts isolated from AIS and LDH patients (n = 3/group). The purity was more than 90%. [file 13018_2022_2978_MOESM1_ESM.tif]

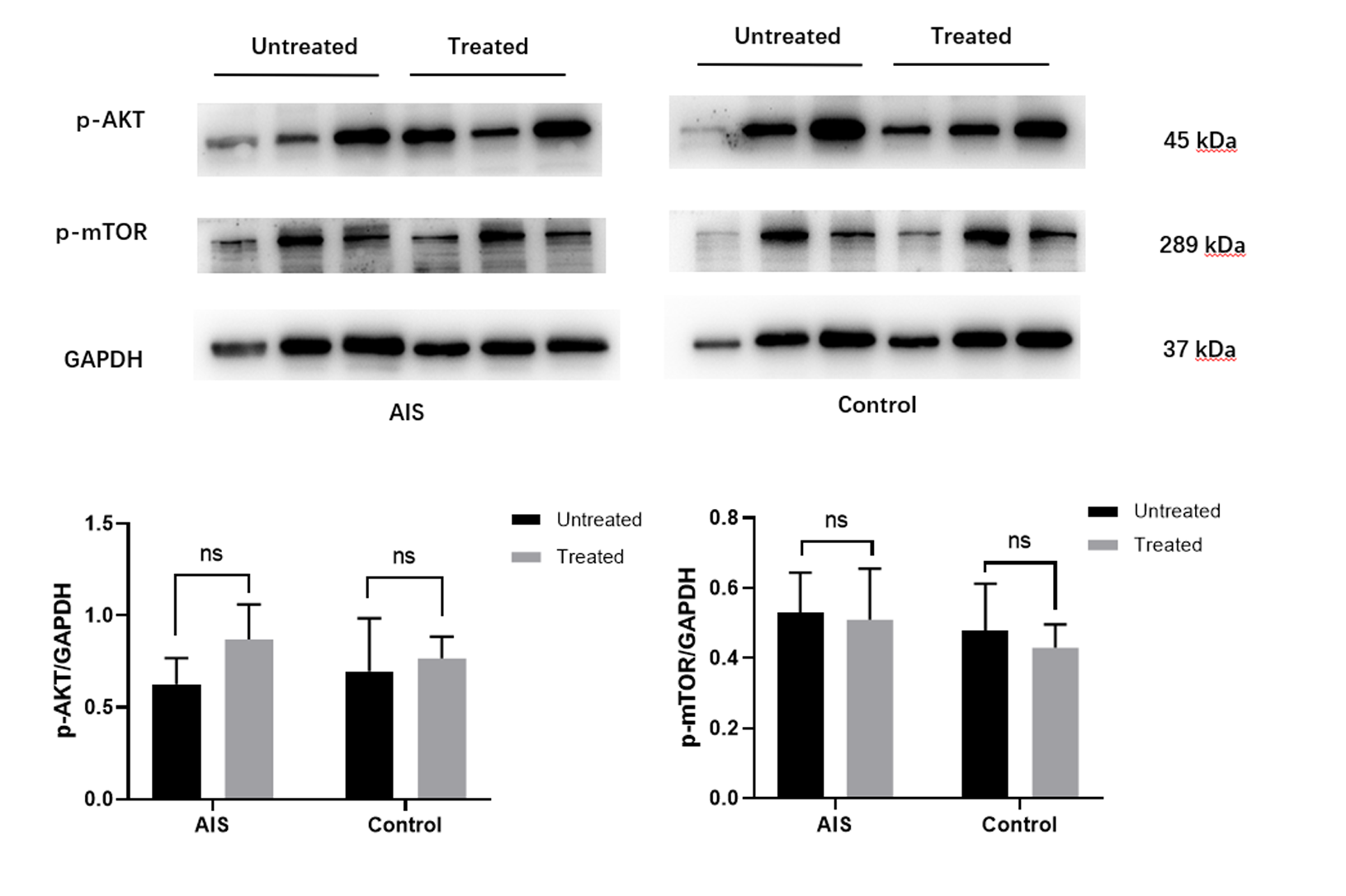

Supplement: Supplementary file 2 — Additional file 2: Fig. S2. Impact of treatment with glucose and insulin on AKT/mTOR pathway. The p-AKT and p-mTOR protein expression was comparable between the untreated and treated group (n = 3/group). ns, not significant. two-tailed paired Student’s t test. Data are presented as mean ± standard deviation. [file 13018_2022_2978_MOESM2_ESM.tif]
